# Did granny know best? Evaluating the antibacterial, antifungal and antiviral efficacy of acetic acid for home care procedures

**DOI:** 10.1186/s12866-020-01948-8

**Published:** 2020-08-26

**Authors:** Marc-Kevin Zinn, Dirk Bockmühl

**Affiliations:** Faculty of Life Sciences, Rhine-Waal University of Applied Sciences, Marie-Curie-Strasse 1, 47533 Kleve, Germany

**Keywords:** Acetic acid, Antimicrobial, Antifungal, Antiviral, Domestic hygiene

## Abstract

**Background:**

Acetic acid has been used to clean and disinfect surfaces in the household for many decades. The antimicrobial efficacy of cleaning procedures can be considered particularly important for young, old, pregnant, immunocompromised people, but may also concern other groups, particularly with regards to the COVID-19 pandemics.

This study aimed to show that acetic acid exhibit an antibacterial and antifungal activity when used for cleaning purposes and is able to destroy certain viruses. Furthermore, a disinfecting effect of laundry in a simulated washing cycle has been investigated.

**Results:**

At a concentration of 10% and in presence of 1.5% citric acid, acetic acid showed a reduction of > 5-log steps according to the specifications of DIN EN 1040 and DIN EN 1275 for the following microorganisms: *P. aeruginosa*, *E. coli*, *S. aureus*, *L. monocytogenes*, *K. pneumoniae*, *E. hirae and A. brasiliensis*. For MRSA a logarithmic reduction of 3.19 was obtained.

Tests on surfaces according to DIN EN 13697 showed a complete reduction (> 5-log steps) for *P. aeruginosa*, *E. coli*, *S. aureus*, *E. hirae*, *A. brasiliensis* and *C. albicans* at an acetic acid concentration of already 5%.

Virucidal efficacy tests according to DIN EN 14476 and DIN EN 16777 showed a reduction of ≥4-log-steps against the Modified Vaccinia virus Ankara (MVA) for acetic acid concentrations of 5% or higher.

The results suggest that acetic acid does not have a disinfecting effect on microorganisms in a dosage that is commonly used for cleaning. However, this can be achieved by increasing the concentration of acetic acid used, especially when combined with citric acid.

**Conclusions:**

Our results show a disinfecting effect of acetic acid in a concentration of 10% and in presence of 1.5% citric acid against a variety of microorganisms. A virucidal effect against enveloped viruses could also be proven. Furthermore, the results showed a considerable antimicrobial effect of acetic acid when used in domestic laundry procedures.

## Background

People have been using natural products like vinegar to clean and sanitize surfaces in the domestic environment for decades [1]. However, there is little scientific evidence on the antimicrobial efficacy of these traditional cleaning methods.

Inter alia, an appropriate, yet effective use of antimicrobial active products must be considered important to prevent the spread of infections. At home, especially young, old, pregnant and immunocompromised persons (YOPIs) are at higher risk. Many potential pathogens such as *Pseudomonas aeruginosa*, members of the *Enterobacteriacea*e family or even methicillin-resistant *Staphylococcus aureus* (MRSA) have already been found to be present on household surfaces [2–6]. In order to achieve an adequate hygiene at home, many people use bleaching agents, as these are readily available, relatively inexpensive and have a very good antimicrobial effect [7–9]. On the other hand, consumers do not want to use “chemical” cleaning agents and thus like to use “green” alternatives such as vinegar. Already in 2000, Rutala et al. were able to show that undiluted white distilled vinegar has a strong effect against *Salmonella spp.* and *P. aeruginosa* at an exposure time of 30 s, but does not work well against *S. aureus* and *Escherichia coli* [10]. Vinegar is mainly comprised of acetic acid, a weak organic acid, for which an antimicrobial effect is mainly delivered by its undissociated form, by passive diffusion through the cell wall of the bacteria. The resulting change of the internal pH is believed to have an inhibitory effect on the bacteria by releasing protons [11].

Acetic acid has already been used in the food industry to inhibit food pathogens. Various studies have shown a protective effect of acetic acid on various types of meat [12], tomatoes [13], carrots [14] and some salads [15]. Further studies were also able to proof an inhibitory effect against certain microorganisms such as *Enterobacteriaceae* [13, 14, 16–18].

Not only bacteria, but also viruses such as the Norovirus, which belongs to the *Caliciviridae* family [19, 20] the annually occurring Influenza virus [21] and above all the new Coronavirus SARS-CoV-2 [22], must be considered important for domestic hygiene procedures. Norovirus is the leading cause of non-bacterial gastroenteritis in both industrialised and developing countries [23]. Here, infection usually occurs via the faecal-oral route, e.g. by ingesting contaminated food or water or via contact to droplets and aerosols of an infected person [24–27]. SARS-CoV-2, as a member of the *Coronaviridae* family, is an enveloped virus, which can cause a severe form of pneumonia and has impacted the global community in an unseen manner since its emergence in December 2019 [28]. Apart from changing the daily life of billions of people, the COVID-19-pandemic has also led to a special perception for the proper inactivation of microorganisms in home care procedures and a fallback to traditional cleaning options with hygienic effects for lack of available disinfectants. As mentioned above, vinegar is widely believed to be an effective means for hygienic cleaning [29, 30]. However, there is little scientific evidence for the antimicrobial efficacy of acetic acid based products for domestic cleaning and laundering.

Hence, the present study aimed to provide data on the antimicrobial efficacy of acetic acid, especially when used in domestic cleaning and laundering procedures. For this purpose, antibacterial, antifungal and antiviral efficacy tests based on existing and adapted standard protocols have been conducted to evaluate the hygienic potential of acetic acid [31–34].

## Results

### Bactericidal and fungicidal activity in suspension tests

**Fig. 1:**
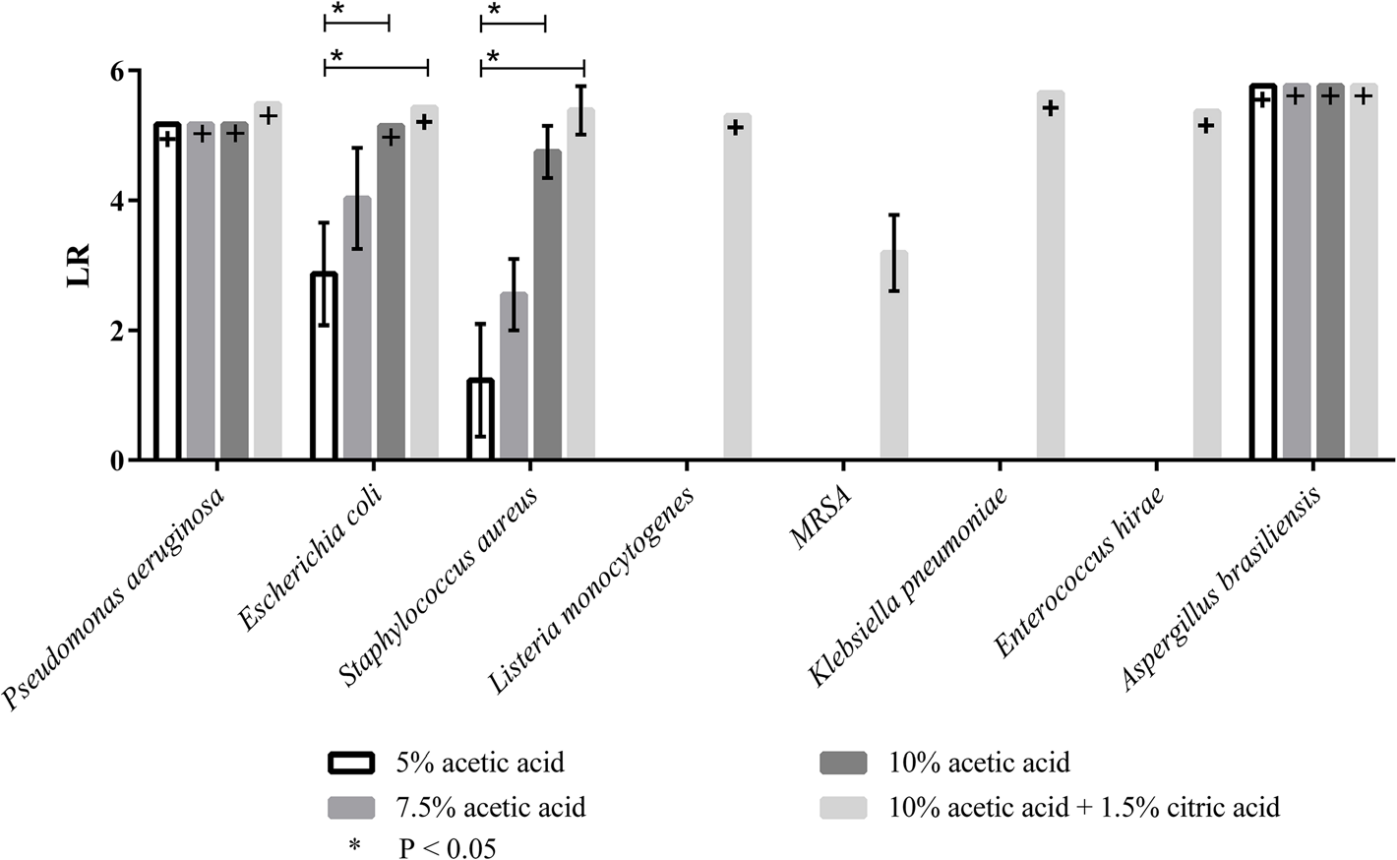
LR for *Pseudomonas aeruginosa, Escherichia coli, Staphylococcus aureus, Aspergillus brasiliensis, Listeria monocytogenes, Methicillin-resistant Staphylococcus aureus (MRSA), Klebsiella pneumoniae* and *Enterococcus hirae* (according to DIN EN 1040:2006–03 and DIN EN 1275:2006–03). The different values for LR max. were obtained due to different initial loads. [+] indicates a complete reduction of the microbial load. (*n* = 3)

To assess its possible use for hygienic cleaning, acetic acid in different concentrations and combined with citric acid, was first evaluated in suspension tests according to DIN EN 1040 and DIN EN 1275. The logarithmic reduction factors (LR) for an extended spectrum of test strains are summarised in Fig. 1.

The results show that acetic acid in all tested concentrations lead to a complete reduction for *P. aeruginosa* and *A. brasiliensis*. For *E. coli,* a complete reduction could be achieved when using 10% acetic acid concentration, either alone or in combination with 2% citric acid. The (LR) of *S. aureus* increased with increasing concentrations of acetic acid and reached a maximum when 10% acetic acid and 2% citric acid was used, without, however, being able to exhibit a complete reduction. Furthermore, a complete reduction was achieved with a test concentration of 10% acetic acid with the addition of 2% citric acid for the microorganisms *L. monocytogenes* and *K. pneumoniae*. For MRSA a maximum reduction of 3.19 was achieved. At an acetic acid concentration of 10%, a complete reduction was achieved for *P. aeruginosa*, *E. coli* and *A. brasiliensis*. For *S aureus* an LR of 4.75 could be detected. At acetic acid concentrations of 7.5 and 5% respectively, no sufficient reductions (LR 4.03 to 1.23) could be achieved for the microorganisms *E. coli* and *S. aureus*.

The microorganisms *L. monocytogenes*, MRSA, *K. pneumoniae* and *E. hirae* were only tested with 10% acetic acid + 1.5% citric acid, as this was the only concentration at which a LR of > 5 log steps was acheived for the other microorganisms.

### Bactericidal and fungicidal activity in surface tests

**Fig. 2:**
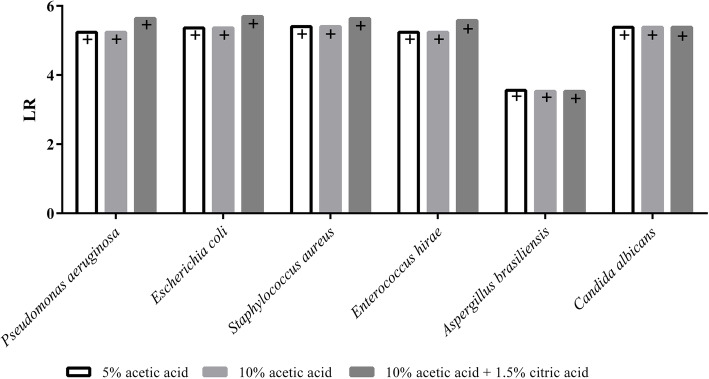
LR for *P. aeruginosa, E .coli, S. aureus, A brasiliensis, C. albicans and E.* hirae (DIN EN 13697:2015–06). The different values for LR max [+] (indicating a complete reduction of the microbial load) were obtained due to different initial loads. (*n* = 3)

The antimicrobial efficacy of 5 and 10% acetic acid as well as a combination of 10% acetic acid and 1.5% citric acid was evaluated on surfaces, since suspension tests are not reflecting this application very well. The logarithmic reduction factors (LR) for an extended spectrum of test strains are summarised in Fig. 2.

The results show that for all tested microorganisms in the three tested concentrations a complete reduction could be demonstrated.

### Virucidal activity

**Fig. 3:**
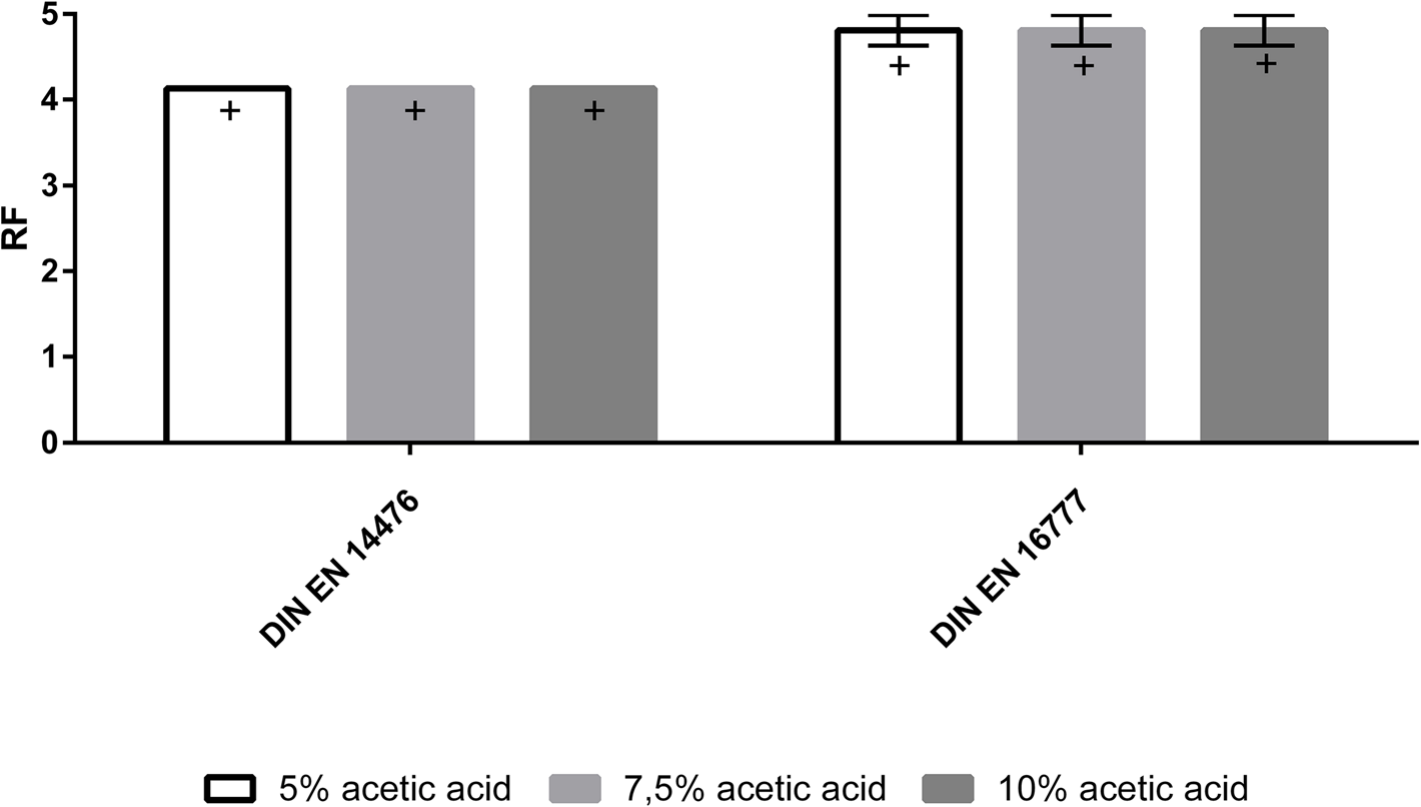
LR for acetic acid concentrations of 5, 7.5 and 10% or 15% according to EN 14476 and EN 16777. All concentrations were tested against the modified vaccinia virus Ankara (MVA) for 1 min. [+] means that the values are ≥ the value shown. (*n* = 2)

In order to test the virucidal activity of acetic acid, the tests were carried out in accordance with the standards EN 14476 [35] and EN 16777 [36], where the effect against the Modified Vaccinia virus Ankara (MVA) was tested in suspension and on surfaces, respectively (Fig. 3).

The results of the virucidal tests show that a complete reduction (≥ 4 log) could be achieved for all tested acetic acid concentrations (5, 7.5 and 10%) after 1 min contact time. According to the standards used (DIN EN 14476 and DIN EN 16777), a product is considered virucidal as soon as it has achieved a reduction of ≥4 log.

### Antibacterial activity in laundering procedures

**Fig. 4:**
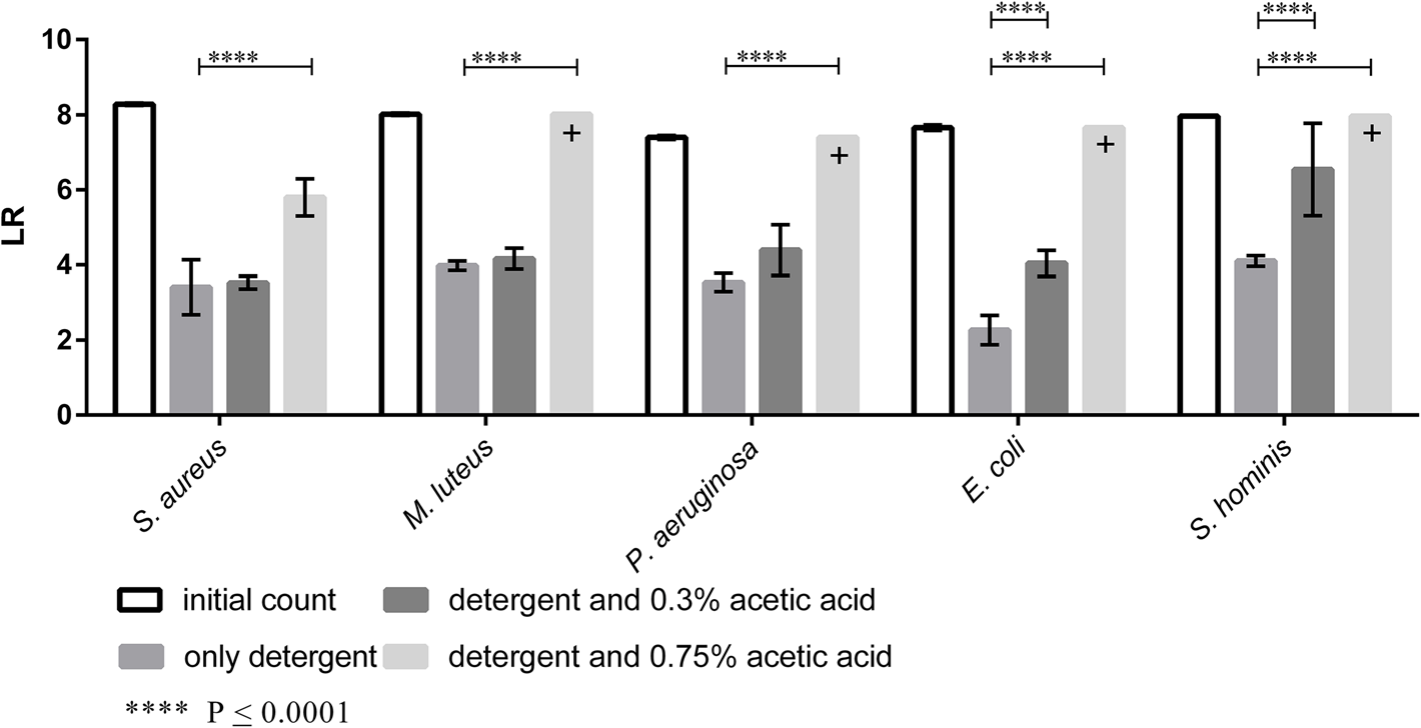
LR of *S. aureus, M. luteus, P. aeruginosa, E. coli* and *S. hominis* after a simulated main wash cycle (60 min at 30 °C) in the Rotawash using liquid detergent. A washing cycle without addition of acetic acid, a wash cycle with addition of 0.3%L acetic acid and a wash cycle using 0.75% acetic acid. The different values for LR max [+] (indicating a complete reduction of the microbial load) were obtained due to different initial loads. (*n* = 3)

To assess a putative effect of acetic acid in a laundry application, the LR of selected microorganisms was determined in a simulated main wash cycle using a lab-scale washing machine (Rotawash). In contrast to the previous tests, a total concentration of 0.3% or 0.75% acetic acid was added to the wash liquor, alongside with a standard laundry detergent. The LR achieved in these tests are shown in Fig. 4.

The results show that for *S. aureus*, *M. luteus* and *P. aeruginosa* there was no significant difference in the LR between a wash cycle where only detergent was used or a cycle where 0.3% acetic acid was added to the wash liquor including the detergent. In contrast, a significant increase of the LR could be demonstrated for the *E. coli* and *S. hominis* when 0.3% acetic acid was added. Furthermore, a significant increase in LR could be observed for all tested microorganisms when 0.75% acetic acid was added to the wash liquor. Here, a complete reduction could be observed for all bacterial test strains, except for *S. aureus*, for which a LR of 5.8 was determined.

## Discussion

The current study aimed to investigate the antimicrobial, antifungal and antiviral effects of acetic acid for domestic cleaning and laundering based on different standard procedures and comprehensive tests. Although there are many studies that have investigated the antibacterial and antifungal effects of acetic acid [15, 37–42] there is no available data on how acetic acid does perform in standard procedures for the testing of disinfectants in suspension or on surfaces. Likewise, the potential of acetic acid for laundry procedures has not been investigated before, although it is known that consumers sometimes use this substance as an additive to increase the hygiene performance of laundering [1]. Finally, it turned out that the COVID-19-pandemic in 2020 lead to an increased demand for pragmatic, yet effective solutions to improve domestic hygiene, particularly with regards to viruses.

The results of this study showed that formulas containing an10% acetic acid and 1.5% citric acid are able to meet the standard requirements for disinfectants (i.e. a LR of > 5), for all tested bacterial and fungal strains except for MRSA, which fits well with the findings of numerous other studies [38–40, 43–47]. In addition to the suspension tests (DIN EN 1040 and DIN EN 1275), DIN EN 13697 was used to test the disinfectant effect on a surface. Here, the results obtained clearly show that a complete reduction could achieved for all tested microorganisms even at lower concentrations of acetic acid.

Ayhan and Bilici could show that acetic acid disrupts the cell wall structure and thus causes a loss of ATP in the cell [43]. Another study suggests that polyphenol compounds may also play a role in the antimicrobial effect of acetic acid. It was proven that polyphenols combine with the peptidoglycan structure of the cell wall and the phospholipid bilayer in the outer membrane of gram negative bacteria and thus impair the integrity of the cell. Furthermore, polyphenols were shown to interfere with the activity of the intracellular bacterial enzymes by inhibiting the formation of amino and carboxyl groups of proteins [44]. This supports the findings that polyphenols present in the acetic acid possess antimicrobial activity against a broad spectrum of microorganisms [48, 49].

Nastou et al. tested the effects of household washing treatments to control *L. monocytogenes* from lettuce. It was shown that application of 1% acetic acid resulted in a reduction of microorganisms by 1 log. According to the results of Nastou et al., which were able to disrupt an inhibitory activity of acetic acid, this effect is proportional to the concentration used [45]. Medina et al. also showed that vinegar (acetic acid) led to a complete reduction of *L. monocytogenes* and killed a high number of *E. coli* and *S. aureus* [39]. These results support the data obtained in this study, as the maximum LR of *L. monocytogenes* of 7.31 was achieved with a 10% acetic acid concentration and a citric acid concentration of 1.5%. Furthermore, LRs of 5.43 and 5.39 for *E. coli* and *S. aureus* were achieved, respectively.

Gopal et al. (2017) showed that an acetic acid concentration of 25% led to a complete reduction of *B. subtilis*, *E. coli* and *P. aeruginosa*. These results are consistent with some pre-tests of the work presented here (data not shown). Furthermore, the study of Gopal et al. indicated that a 10% acetic acid led to a complete reduction for *Aspergillus niger* (now: *A. brasiliensis* [50]) and a reduction of more than 2 log steps for *Candida albicans* (*C. albicans*) [40]. The results obtained in the present study largely agree with these findings. The study at hand also obtained a complete reduction for *A. brasiliensis* already at an acetic acid concentration of 5%. The results for *C. albicans*, however, are different in the current study, since were also able to achieve a complete reduction of *C. albicans* at a low acetic acid concentrations (5%). These differences might be explained by the vinegar used, since Gopal et al. used an apple cider vinegar, whereas in the current study vinegar made from acetic acid diluted with water and purified to a high degree of purity was used.

Ryssel et al. investigated whether acetic acid might be used as an alternative for common local antiseptics. They mixed 0.1 mL bacteria solution (bacterial count approx. 10^7^–10^8^ cfu/mL) with 9.9 mL acetic acid (3%) and incubated the mixture for 5, 30 and 60 min at a temperature of 37 °C. They showed that at 3% acetic acid concentration no colonies of *P. aeruginosa*, of *P. vulgaris*, of *A. baumannii* and of β-haemolytic Group B *Streptococci* could be detected after 5 min incubation. Furthermore *E. coli*, *E. faecalis* and MRSA were eliminated after a exposure time of 60 min [47]. Our experimental design used an incubation time of 5 min and also showed a complete reduction of *P. aeruginosa*. In contrast to the attempt of Ryssel et al. the current study also tested up to a concentration that would be required to pass disinfection tests, which for most observed microorganisms was 10% acetic acid and 1.5% citric acid. At this concentration, a complete reduction for *E. coli* and a LR of 3.19 for MRSA could already be demonstrated with an incubation time of 5 min.

Overall, there has been little research in the literature on the virucidal effect of acetic acid against enveloped viruses. In 2005, Rabenau et al. investigated the stability and inactivation of the SARS coronavirus and could show that an acetic acid concentration of 6% leads to a reduction of > 3 log levels within 60 s [51]. In contrast to the present study the authors aimed to investigate the stability of the SARS coronavirus. Nevertheless, the data of Rabenau et al. confirm the current results, which suggest a complete reduction against enveloped viruses (see Fig. 3) at a concentration of 5%. In 2010 Greatorex et al. were able to show in a study that acetic acid in a concentration of 10% is effective against the influenza virus A/H1N1 [52]. This result agrees with those of the present study, which showed that acetic acid is effective against the MVA at a concentration of 5%. This could be demonstrated on the basis of the standards DIN EN 14476 and DIN EN 16777, which apply to disinfectant tests with regard to virucidal activity. The present study could confirm the virucidal effect of acetic acid on the basis of existing standards. However, no tests were carried out on the virucidal effect in washing machines, because Heinzel et al. were able to show in 2010 that conventional household washing detergents achieve a complete reduction of enveloped and non-enveloped viruses already at 40 °C [53]. The acetic acid concentrations tested in the present study were chosen based on the results of antibacterial and antifungal tests. As the results suggest that acetic acid concentrations of 10% + 1.5% citric acid showed the highest reductions, the tested concentrations (5, 7.5 and 10% acetic acid) were taken for the virucidal activity tests.

The results of the simulated washing process using the Rotawash showed that a complete reduction of four microorganisms (*M. luteus*, *P. aeruginosa*, *E. coli* and *S. hominis*) could be achieved by adding an acetic acid concentration of 0.75% to the wash liquor. Likewise, a high LR of 5.8 could be achieved for *S. aureus*. Thus, a disinfecting effect of acetic acid was proven for all tested microorganisms at an effective concentration of 0.75% acetic acid. The acetic acid concentration of 0.3% was used since it corresponds approximately to the dosage of commercially available laundry sanitizers [54]. Assuming that a common washing machine uses approx. 10 L of water for each wash step, a final concentration of 0.3% would equal a dosage of 120 mL of a commercially available vinegar essence containing 25% acetic acid. Consequently, a final concentration of 0.75% would require the use of 300 mL vinegar essence, which is still in the range that can be considered to be applied by consumers. The results showed that for *S. aureus* and *M. luteus* no additional antimicrobial effect was detected for the lower concentration of acetic acid compared to a simulated wash cycle with detergent alone. However, a significant difference (2- way- ANOVA) for an additional dosage of 0.3% acetic acid could be demonstrated for *E. coli* and *S. hominis*, which also exhibits a disinfecting effect with an LR of 6.5. These findings suggest that a considerable antibacterial effect may be expected, when acetic acid is used a hygiene additive for laundry.

## Conclusion

The results of this study show that acetic acid in a concentration of 10% and an addition of 1.5% citric acid has a disinfecting effect against a variety of microorganisms. In addition to the typical pathogens *E. coli*, *S. aureus* and *L. monocytogenes*, also *P. aeruginosa*, *K. pneumoniae*, *E. hirae*, *A. brasiliensis* and *C. albicans* are among the microorganisms that achieve a reduction of > 5-log steps against acetic acid in the concentration mentioned. Furthermore, this study was able to show that acetic acid in a concentration of 5, 7.5 and 10% is also effective against enveloped viruses.

Moreover the present study showed that acetic acid above a certain concentration also has disinfecting properties on the laundry in a washing machine. It could be shown that an above-average dosage of the acetic acid *S. aureus*, *M. luteus*, *P. aeruginosa*, *E. coli* and *S. hominis* > 5 log- steps are reduced.

## Methods

### Determination of the bactericidal, fungicidal and virucidal activity in suspension tests

The suspension tests were performed according to the standards DIN EN 1040:2006 and DIN EN 1275:2006 [31, 32] for bacteria and fungi and to the standard DIN EN 14476 [35] for viruses. All tests were performed under clean conditions, i.e. in presence of an organic challenge (0.3 g/ L albumine) at room temperature. As specified in the standards, the contact time was 5 min for bacteria and 15 min for yeasts. Likewise, the microorganisms suspension used ranged between 1.5 and 5 × 10^8^ cfu/mL. Unlike described in DIN EN 1040 and DIN EN 1275 1 mL was used in the experiments instead of 10 mL. All products were tested at room temperature. Neutralization was carried out by dilution using an inactivator solution comprised of Tween 80 (30 g/L), Lecithin (3 g/L), L-histidine (1 g/L), Sodium-thiosulfate (5 g/L) and Saponin (30 g/L). The virucidal tests were carried out strictly according to DIN EN 14476. The calculation of the reduction factors was done as described in chapter ‘Microbiological and statistical analysis’.

All strains were purchased at the German Collection of Microorganisms and Cell Cultures (DSMZ, Brunswick, Germany), except from the Methicillin resistant *Staphylococcus aureus* (MRSA), which was derived from the Culture Collection of the University of Gothenburg (CCUG). The corresponding strain code of the American Type Culture Collection (ATCC) is provided in Table 1 for information only.

**Table 1:** Fungal, bacterial and viral test strains

Strain	Code
**Fungal strains:**	
*Aspergillus brasiliensis*	DSM 1387, ATCC 16404
*Candida albicans*	DSM 1386, ATCC 10231
**Bacterial strains:**	
*Enterococcus hirae*	DSM 3320, ATCC 10541
*Escherichia coli*	DSM 682, ATCC 10536
*Klebsiella pneumoniae*	DSM 26371, ATCC 700603
*Listeria monocytogenes*	DSM 20600, ATCC 15313
*Micrococcus luteus*	DSM 1790, ATCC 10240
*Pseudomonas aeruginosa*	DSM 939, ATCC 15442
*Staphylococcus aureus*	DSM 799, ATCC 6538
*Staphylococcus aureus, Methicillin resistant*	CCUG 35601
*cStaphylococcus hominis*	DSM 20329, ATCC 27845
**Viral strains:**	
Modified Vaccinia Ankara virus (MVA)	ATCC VR-1508

### Determination of the bactericidal, fungicidal and virucidal activity in surface tests

The determination of bactericidal an fungicidal activity on surfaces was performed according to DIN EN 13697 [33] and for virucidal activity according DIN EN 16777 [36]. All tests using bacterial strains were executed at Rhine-Waal-University of Applied Sciences; for virucidal tests, an external lab (Dr. Brill und Dr. Steinmann Institute for Hygiene and Microbiology, Hamburg, Germany) was commissioned by the funder of this study.

All tests were performed under clean conditions, i.e. in presence of an organic challenge (0.3 g/L albumine) at room temperature. As specified in the standards, the contact time was 5 min for bacteria and 15 min for yeasts. Likewise, the microorganisms suspension used ranged between 1.5 and 5 × 10^8^ cfu/mL. For the tests according to DIN EN 13697 *P. aeruginosa*, *E. coli*, *S. aureus*, *E. hirae*, *A. brasiliensis* and *C. albicans* were used; for the tests according to DIN EN 16777 the Modified Vaccinia Ankara virus (MVA) was used. The calculations of the reduction factors were performed as described in chapter ‘Microbiological and statistical analysis’.

### Determination of the antibacterial activity in laundering procedures using a laboratory washing machine

To assess the antimicrobial performance of products containing acetic acid for use in laundry detergents, a laboratory washing machine (Rotawash M228C, SDL Atlas, Rock Hill, SC, USA)) was used as described in Schages et al. [34]. To simulate a normal household washing machine all quantities were downscaled adequately, i.e. a 1 L vessel was filled with 0.5 L of water in addition to the ballast load textiles, the soil ballast, the detergent and eight steel beads (to simulate the mechanics of a washing machine) as described below.

In this study, cotton (wfk 10 A, 170 g/m^2^, wfk testgewebe, Brüggen, Germany) was used as the ballast load. In addition to the ballast load, SBL2004 (SBL2004, wfk testgewebe, Brüggen, Germany) was used as a source of organic soil. All materials used are calculated based on the volume of water in a vessel of the laboratory washing machine:

Ballast load (100 g/vessel) consisted of 96.5 g textile ballast of standard cotton and of 3.5 g textile comprised by the SBL2004 swatches equalling approx. 1.2 g standard soil. A liquid heavy duty detergent (Ariel Actilift, Procter & Gamble, Germany) was dosed according to the detergent manufacturers’ instructions (120 mL/10 L) and adjusted to the volume of one vessel of the Rotawash (6 mL/0.5 L).

The duration of the wash cycle in the Rotawash was 60 min, and the temperature was adjusted to 30 °C, at a water inlet temperature of approx. 15 °C – 20 °C. In every test run five artificially contaminated biomonitors (one swatch per microorganism) are added to one vessel. In this series of experiments *S. aureus*, *M. luteus, P. aeruginosa, E. coli* and *S. hominis* were tested. All tests in the Rotawash were run in triplicates. The evaluation is performed as described in chapter ‘Microbiological and statistical analysis’.

### Microbiological and statistical analysis

The microbial count on each contaminated biomonitor was quantified by extraction with 1 mL TSB-TLH-thio (Tryptic Soy Broth with 30 g/L Tween 80, 0.3 g/L lecithin, 1 g/L histidine, 5 g/L sodium-thiosulfate) followed by investigating the colony forming units (cfu/mL) on surface culture on Tryptic Soy Agar (TSA) (Oxoid, Wesel, Germany; incubation at 37 °C for 24 h). Rotawash-tests were carried out in a 1.5 mL reaction tube (Sarstedt, Nürmbrecht, Germany) for 10 min at 15 °C and 1000 rpm in an orbital incubating shaker (Thermomix comfort, Eppendorf, Hamburg, Germany).

The colony forming units (cfu/mL) were investigated in surface culture either on TSA for bacteria (incubation at 37 °C for 24 h) or Malt Extract Agar (MEA) for *C. albicans* and *A. brasiliensis* (incubation at 30 °C for 48 h). After laundering, the microbial count on the test swatches is determined similarly. The number of colony forming units (cfu/mL) on plates was used to calculate the microbial load in the extraction liquid (c_wei_) (Eq. (1)):

1 $$ {C}_{wei}=\frac{\sum C}{\left({n}_1\ast 1\right)+\left({n}_2\ast 0.1\right)}\ast d $$

*C*_*wei*_ = weighted arithmetic average.

∑C = sum of viable cell count of all agar plates, used for calculation.

n_1_ = count of agar plates with the lowest evaluable dilution.

n_2_ = count of agar plates of the next higher dilution stage.

d = dilution factor of the lowest evaluable dilution stage.

Plates with less than 10 cfu or more than 300 cfu were not considered.

To calculate the LR, the logarithmic cfu value of the biomonitors was subtracted from the logarithmic mean of the initial microbial count of the respective biomonitors.

2 $$ LR={K}_0-{K}_S $$

LR = logarithmic reduction factor.

K_0_ = common logarithmic of the microbial count per mL of the initial load on the swatches before laundering.

K_S_ = common logarithmic of the microbial count per mL of the initial load on the swatches after laundering.

Unless otherwise stated, the tests were performed in triplicates and statistically evaluated in the case of a non-Gaussian distribution using Students *t*-test, Kruskal-Wallis or in the case of a Gaussian distribution using a 2-way ANOVA.

## Data Availability

The datasets used and/or analysed during the current study are available from the corresponding author on reasonable request.

## Abbreviations

*A. brasiliensis*: *Aspergillus brasiliensis*
*C. albicans*: *Candida albicans*
*E. hirae*: *Enterococcus hirae*
*E. coli*: *Escherichia coli*
*K. pneumoniae*: *Klebsiella pneumoniae*
*L. monocytogenes*: *Listeria monocytogenes*
LR: Logarithmic reduction factor
MEA: Malt Extract Agar
*M. luteus*: *Micrococcus luteus*
MRSA: *Methicilin resistant Staphylococcus aureus*
MVA: Modified Vaccinia Ankara virus
*P. aeruginosa*: *Pseudomonas aeruginosa*
*S. aureus*: *Staphylococcus aureus*
*S. hominis*: *Staphylococcus hominis*
TSA: Tryptic Soy Agar
TSB: Tryptic Soy Broth
